# Overall grazing tolerance index (overall GTI) is not an ideal predictor for describing a single‐species tolerance to grazing

**DOI:** 10.1002/ece3.5038

**Published:** 2019-03-12

**Authors:** Lulu Zhang, Zhihong Zhu, Yingnian Li, Zengqiang Qian, Gang Liu, Xiaoan Wang

**Affiliations:** ^1^ College of Life Sciences Shaanxi Normal University Xi'an China; ^2^ Northwest Institute of Plateau Biology Chinese Academy of Sciences Xining China

**Keywords:** alpine meadow, clipping, effective predictor, *Elymus nutans*, tolerance expression

## Abstract

Plants' pattern of compensatory growth is often used to intuitively estimate their grazing tolerance. However, this tolerance is sometimes measured by the overall grazing tolerance index (overall GTI), which assumes that tolerance is a multivariate linear function of various underlying mechanisms. Because the interaction among mechanisms is not independent, the grazing tolerance expression based on overall GTI may be inconsistent with that based on compensatory growth. Through a manipulative field experiment from 2007 to 2012, we measured the responses of 12 traits of *Elymus nutans* to clipping under different resource availabilities in an alpine meadow and explored the compensatory aboveground biomass and the overall GTI to assess the possible differences between the two expressions of tolerance. Our results showed that these two expressions of tolerance were completely opposite. The expression based on overall GTI was over‐compensatory and did not vary with clipping and resource availability, while the expression based on compensatory aboveground biomass was under‐compensatory and altered to over‐compensation after fertilization. The over‐expression of highly variable traits with extremely high negative mean GTI to defoliation damage, the influence of random errors contained in traits considered, and the doubling weight of functional redundant traits greatly inflated the overall GTI, which leads to the inconsistency of the two tolerance expressions. This inconsistency is also associated with the different determining mechanisms of the two tolerance expressions. Our data suggest that plants' grazing tolerance is not a multivariate linear function of traits or mechanisms that determine grazing tolerance; the overall GTI is only a measure of traits' variability to defoliation damage. Our findings highlight that the tolerance of *E. nutans *mainly depends on the response of traits with lower variability to defoliation, and the overall GTI is not an ideal predictor for describing a single‐species tolerance to grazing.

## 1 INTRODUCTION

The interactions between plants and herbivores are among the most important ecological interactions in nature (Johnson, 2011). In grassland ecosystems, many plants tend to tolerate rather than resist the loss of aboveground tissues by way of compensatory growth following grazing (Westoby, 1989). Tolerance refers the ability of plants to regrow and/or reproduce after herbivory, which has traditionally been characterized by a single trait and estimated by the either the difference in fitness between related damaged and undamaged plants or the proportional fitness of damaged individuals relative to undamaged ones (Strauss & Agrawal, 1999). Compensatory growth is a classical measure of plant grazing tolerance (Leriche et al., 2003), commonly defined as a positive response of plants to injury, which has been used to describe plant responses ranging from a partial replacement of lost tissue to a net productivity exceeding that of uninjured control plants (Belsky, 1986), which included under‐compensate, equally compensate, or over‐compensate (Westoby, 1989). In generally, defoliation can promote growth by five mechanisms, such as stimulating photosynthesis, removing old and dead tissue, altering mass allocation, increasing growth rate, and producing more reproductive tillers in defoliated plants, and finally shown in the changes of aboveground biomass (Hilbert, Swift, Detling, & Dyer, 1981; Oesterheld & McNaughton, 1991; Zhao, Chen, & Lin, 2008). Thus, the changes of aboveground biomass are the most intuitive expression of changes in traits or underlying mechanisms; by estimating the compensatory growth pattern, we can easily and intuitively determine the grazing tolerance of a particular species. This concept has been widely employed by relevant studies (Anten, Martínez‐Ramos, & Ackerly, 2003; Belsky, 1986; Kohyani, Bossuyt, Bonte, & Hoffmann, 2009; Suwa & Maherali, 2008). In fact, tolerance or the amount of compensatory growth is generally the result of diverse plant responses and life histories (Strauss & Agrawal, 1999; Tiffin, 2000) or the combined action of several different traits (Wise, Cummins, & De Young, 2008). Perhaps for this reason, Damhoureyeh and Hartnett (2002) used a multitrait metric, overall grazing tolerance index (overall GTI), to compare the variation in grazing tolerance and mechanisms among three tallgrass prairie plant species. Since then, however, this multitrait index has not been widely used by other researchers, nor has it been reported on its applicability.

The overall GTI of a plant species is expressed as a mean percent reduction in overall species' grazing tolerance performance, which equal to the arithmetic mean of the percent reductions of traits considered (Damhoureyeh & Hartnett, 2002). The percent reduction (or GTI) of a given trait equals the difference between the induced value after defoliation and the initial value before defoliation as a percentage of the initial value (see Formula (4) for this article). It is obvious that the GTI is strongly affected by the variability of a trait to defoliation damage in response, because relative to the initial value, either a larger or a smaller induced value indicates that the trait is highly variable to defoliation damage. In addition, the overall GTI calculated from the arithmetic mean of GTI for several traits considered presupposes that the performance of these traits has an additive effect on plant growth. Therefore, we believe that the concept of overall GTI actually implies two assumptions: (a) The tolerance of plants is a measure of traits' variability to defoliation damage; that is, the greater the induced value is relative to the initial value, the more tolerance will be increased, and otherwise, the more the tolerance will be reduced. And (b) the overall GTI of a species is the multivariate linear function of the GTI of traits or mechanisms considered. However, the variability or range of each trait is relatively fixed due to phylogenetic constraints and varies greatly from trait‐to‐trait (Pérez‐Harguindeguy et al., 2013), the increase in some highly variable traits (e.g., the relative growth rate) does not necessarily cause an increase in plant biomass after grazing (Hilbert et al., 1981; Oesterheld & McNaughton, 1991; Tiffin, 2000; Zhao et al., 2008), and the interactions between the various potential mechanisms may not be independent after defoliation damage (Lepš, Bello, Lavorel, & Berman, 2006; Tiffin, 2000); that is, the effects of these traits or mechanisms on grazing tolerance might be nonadditive. If this is the case, then the two expressions of grazing tolerance based on overall GTI and compensatory growth may be inconsistent. Therefore, the simplest way to evaluate whether the overall GTI is a valuable indicator of grazing tolerance is to test whether the two expressions of the same species after defoliation damage are consistent. If the expression pattern based on overall GTI is inconsistent with the classical expression pattern based on compensatory growth, we can conclude that the overall GTI may not be an ideal estimator for evaluating compensatory growth capacity.

**Figure 1:**
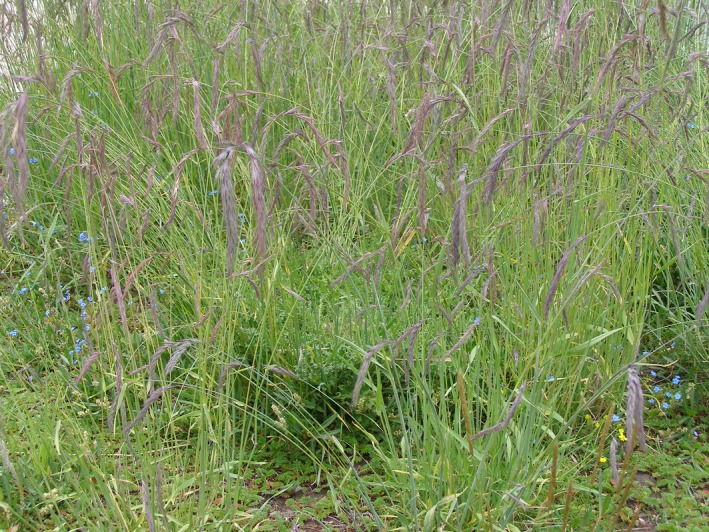
*Elymus nutans* Griseb., a perennial herb belonging to Gramineae, is widely distributed in Qinghai‐Tibet Plateau and often used to establish artificial or semi‐artificial grassland. This photograph was taken in 2008 by the author of this paper (Zhi‐hong Zhu) in our experimental plots

Here, we measured the response of 12 morphological and physiological traits to clipping under different resource availabilities in *Elymus nutans* (Figure 1), a perennial grass of alpine meadow in the Qinghai‐Tibetan Plateau, through a 6‐year field manipulative experiment that allowed us to examine the effects of repeated defoliation over a long time period and to assess the applicability of overall GTI by testing two underlying assumptions in overall GTI. Firstly, if plant grazing tolerance is a multivariate linear function of traits considered, we expect that the two expressions of grazing tolerance would have the same pattern. Secondly, if grazing tolerance is a measure of the trait' variability to defoliation damage, then the highly variable traits would be effective predictors of compensatory growth and the increase of induced value of these traits should have a great relative contribution to compensatory growth capacity.

## 2 MATERIALS AND METHODS

### 2.1 Study site and species description

This research was carried out in a *Kobresia humilis* meadow of the Haibei National Field Research Station of Alpine Grassland Ecosystem from 2007 to 2012. The location is in the northeast Qinghai‐Tibetan Plateau in a broad NW–SE‐oriented valley surrounded by the Qilian Mountains with a latitude range of 37°29′–37°45′N and a longitude range of 101°12′–101°23′E (Li, Zhao, Cao, Zhao, & Wang, 2004). The altitude of the area ranges from 3,200 to 3,600 m, and the annual average temperature is −1.7°C; the annual average precipitation is 562 mm, of which 80% falls during the growing season from May to September (Li et al., 2004). The annual precipitation from 2007 to 2012 was 510, 429.9, 494.6, 493.3, 375.2, and 352.6 mm, respectively. The average of 442.6 mm was 21.2% lower than the long‐term average annual precipitation (562 mm). *K*. *humilis* meadow, widely distributed in this region, is traditionally grazed by livestock during the winter–spring months from 1 November each year to 31 May of the next year (Li et al., 2004).

*Elymus nutans*, a perennial grass with short rhizomes and adult plant heights of 60–150 cm, is a common dominant species of the meadow (Lu & Nie, 2002) and was often used to establish artificial or semi‐artificial grasslands in the Qinghai‐Tibet Plateau over the past years due to its rapid growth and high aboveground biomass production (Feng et al., 2010). However, due to the sharp decline in regrowth and seed production caused by livestock heavy grazing, these grasslands inevitably degraded in the years following their establishment (Liu et al., 2009; Liu, Zhu, & Zheng, 2005; Wang, Du, & Ren, 2003; Zhu, Liu, & Zheng, 2005). Although fertilization can improve the biomass production of the species (Liu et al., 2005) and prolong the utilization period of the grassland (Wang et al., 2003), the species diversity of grasslands was reduced significantly (Yang, van Ruijven, & Du, 2011). This indicated that the grazing tolerance of this species was lower, but it would increase under the condition of high nutrient supply. Therefore, the accurate assessment of the grazing tolerance of *E. nutans* and its decision mechanisms under various conditions of resource availabilities are essential for the improvement of artificial grassland management and biodiversity protection.

### 2.2 Experimental design

**Figure 2:**
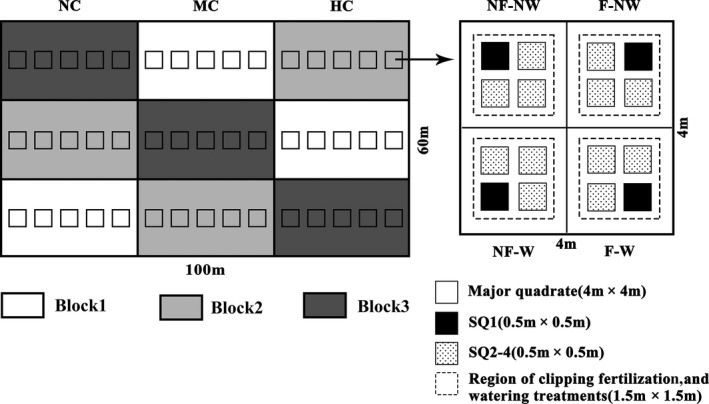
A diagram of the design layout of complete experimental design and four subquadrates within each major quadrate. The experimental plot is 100 m × 60 m. The major quadrate is 4 m × 4 m in size. NF–NW, F–NW, NF–W, and F–W, respectively, correspond to the different treatments of the four subquadrates, that is, (1) neither fertilization nor watering (NF–NW), (2) fertilization but no watering (F–NW), (3) no fertilization but watering (NF–W), and (4) both fertilization and watering (F–W). SQ1–SQ4 represent four small quadrates of 0.5 m × 0.5 m area in each subquadrate, which are used to collect data. The dotted square represents the areas 1.5 m × 1.5 m in size where clipping, fertilization, and watering were performed

Fenced experimental plots (100 × 60 m) were established in early April 2007. For the 15 years prior to the beginning of this study, this experimental plots had been freely grazed during the winter–spring months, with moderate grazing intensity and no fertilization or watering (for details, see Zhu, Wang, & Zhao, 1994; Zhu & Wang, 1996; Zhu & Sun, 1996). Our experiment used a split‐plot design with clipping intensity as the whole plot (including three clipping levels: the heavy clipping [HC], the moderate clipping [MC], and no clipping [NC]) and assigned both fertilizer (two levels: fertilization [F] and no fertilization [NF]) and watering [two levels: watering [W] and no watering [NW]) as the subplots (Figure 2). Three duplicated blocks were set up, and each block included fifteen major quadrates of 4 × 4 m area (each clipping level arranged randomly within each block contains five major quadrates), which was further subdivided into four 2 × 2 m subquadrates by galvanized sheets of iron (2 m in length, 0.25 m in width, and 1.2 mm in thickness), as a total of 45 major quadrates and 180 subquadrates. These sheets of iron were embedded into the soil for 0.25 m in depth to prevent water and fertilizer penetration among subquadrates in soil. At the subplot scale, the design was a factorial combination of fertilization and watering. Clipping, fertilizing, and watering manipulation were applied to the central 1.5 × 1.5 m area of each subquadrate. To avoid edge effects and collect data, we set four 0.5 × 0.5 m small quadrates (i.e., SQ1–SQ4) within each subquadrate, with a 0.2 m spacing distance between them. SQ1 was used to record the ramet density, ramet height, and aboveground biomass of the ramet, and SQ2, SQ3, and SQ4 were used to collect other data (Figure 2).

Clipping was conducted in mid‐June each year during the study period, including 60%–70% (HC), 45%–50% (MC) and 0% (NC) of aerial parts were removed with scissors. After the clipping, about 1 cm and 3 cm height of plants were remnant for treatment HC and MC, respectively. The clipping treatments were designed based on the results of our previous livestock grazing experiment (Zhu & Wang, 1996; Zhu et al., 1994; Zhu, Wang, Li, Wang, & Guo, 2012). Urea 4.60 g/m^2^ (containing N: 20.4%) and diammonium phosphate 1.10 g/m^2^ (containing N: 5.9%, P: 28.0%) were sprinkled onto each fertilized subquadrate three times each year, in the middle of May, June, and July; the addition rates (g m^−2^ year^−1^) corresponded to 3.01 g N and 0.92 g P, respectively. Generally, the input rate of exogenous nitrogen in the region was approximately 3.12–3.63 g N·m^−2^·year^−1^, which including of 2.25 g N m^−2^ year^−1^ application when establishing artificial grassland (Qiao et al., 2006) and of 0.87–1.38 g N m^−2^ year^−1^ atmospheric nitrogen deposition (Lü & Tian, 2007). Our nitrogen addition was slightly lower than the exogenous nitrogen input. After each fertilizing, spray pot was used to add 4.5 kg/m^2^ of water to the subquadrates to be watered. The total amount of water added is 13.5 kg m^−2^ year^−1^, which corresponded to an increase of approximately 3.1% of the average annual precipitation in the area from 2007 to 2012.

Before the first clipping and fertilization were carried out in this study, we conducted a community survey in early May 2007 on the coverage of community, the plant height, the ramet density, and the ramet aboveground biomass of *E*. *nutans*, and also the content of NO_3_^−^, NH_4_^+^, and available phosphorus in soil. The results of mixed‐effects model using three‐way split‐plot ANOVAs showed that there was no significant difference in these measures among different treatments (*p* > 0.05).

### 2.3 Sampling and data collection

Before clipping in mid‐June of each year from 2007 to 2012, the ramet density and height per SQ1 were recorded in situ. Average ramet height was measured randomly on the basis of twenty individual plants or, if the number of existing plants per SQ1 was <20, on the basis of their actual number. Aerial parts of all ramets per SQ1 of the two clipped treatments were cut off by scissors considering the stubble height. At the end of August of each year, the ramet height and density of all SQ1 included unclipped treatment were measured by the same method, and then harvested to estimate the compensatory growth ability.

In order to estimate the compensatory growth ability of aboveground biomass, from 2010 to 2012, five adult ramets were randomly selected in SQ2, SQ3, and SQ4 in the mid‐June of each year; the removed biomass of each ramet was collected and measured. At the end of August, the aboveground biomass of each of five ramets in these small quadrates was harvested. These collected and harvested parts were kept in an oven at 60°C for 72 hr and weighed (0.001 g).

The related calculations of the compensatory growth ability (CGA) and the relative growth rate of height (RGR_H_) during the period were as follows:

(1) $${\text{CGA}}_{x}=\text{clipped measure}\;x\;\text{in June}+\text{harvested measure}\;x\;\text{in August}$$

(2) $${\text{RGR}}_{H}=\left(\text{harvested height in August}-\text{the remaining height in June}\right)/\left(T\times \text{the remaining height in June}\right)$$

where in formula (1) subscript *x* represents ramet height (*H*) or aboveground biomass of ramet (AB), and *T* refers to the number of days after clipping in mid‐June until the end of August. In formula (2) (Ruiz‐R, Ward, & Saltz, 2008), the remaining height of ramet in unclipped treatment was the actual height measured in June because it was not clipped in that time (i.e., the removal height is equal to zero). For ramet clipped in June, the remaining height was the ramet height of reserved part after clipping. We calculated CGA*_H_* (cm), CGA_AB _(g), and RGR_H_ (cm cm^−1^ day^−1^), respectively. CGA_D _(ramets/m^2^) was the compensatory growth ability of ramet density calculated only by ramet density harvested in August.

From 2008 to 2009, we selected three ramets randomly in SQ2, SQ3, and SQ4 in mid‐July (1 month after clipping) and measured the net photosynthetic rate (NPR, µmol m^−2^ s^−1^) of the three youngest, fully mature green leaves per ramet under ambient CO_2_ concentrations with TPS‐I photosynthesis equipment (PP Systems, Ayrshire, UK) from 9:30 to 11:30 on a sunny day.

In mid‐August 2010 and 2012, we selected five adult ramets randomly in SQ2, SQ3, and SQ4, counted the number of healthy leaves without obvious symptoms of pathogen or insect attack per ramet, cut them off, wrapped with moist paper, and put in sealed plastic bags. The total leaf area per ramet was measured with a Handheld Laser Leaf Area Meter (CI‐203, CID, Inc., USA) in the laboratory on the same day to calculate the leaf area (LA, cm^2^). After that, the samples were kept in an oven at 60°C for 72 hr and weighed to calculate the specific leaf area (SLA, cm^2^/g).

In 2009, to estimate the changes in total nonstructural carbohydrates (TNC) in roots, nitrogen contents (N) in leaves and culms and biomass allocation of different organs induced by treatments, we randomly dug out five ramets of different genets in the 0.2‐m spacer region surrounding the four small quadrates of the first two major quadrates in two blocks after clipping about every 10 days. The sampling dates were June 18, June 29, July 11, July 20, July 31, August 13, and August 23. Each ramet was divided into three functional parts: growth organ (incl. leaves and culms, GO), sexual reproductive organ (all inflorescences, RO), and storage organ (i.e., roots and rhizomes, SO). Then, we rinsed the roots of each ramet in running water using a fine mesh sieve (0.2 mm) to remove fine sand and contaminants. After that, different plant parts were kept in an oven at 60°C for 72 hr and weighed (0.001 g). The dried storage organ and growth organ were separately milled and sieved through a 0.15‐mm sieve to a homogeneous powder, and then, TNC of storage organ were determined by the colorimetric anthrone method (Shanghai Institute of Plant Physiology‐Chinese Academy of Science & Shanghai Society of Plant Physiology, 1999) and total N (%) of growth organ was determined by the UDK152 Kieldahl Azotometer (VELP, Inc., Italy). The TNC (mg/g) were obtained using the following formula:

(3) $$\text{TNC}=\left(C\times V_{\text{t}}\times n\right)/\left(W\times V_{\text{s}}\right)$$

where *C* is the sample concentration of the standard curve (mg), *V*_t_ is the solution volume (ml), *n* is the sample dilution ratio, *W* is the sample mass (g), and *V*_s_ is the sampling volume (ml).

The dry mass of different organs per ramet was used to calculate the biomass allocation parameters. The root/shoot ratio (R/S ratio) is the ratio of root biomass to aboveground biomass per ramet. Sexual reproductive allocation (SRA), growth allocation (GA), and storage allocation (SA) were expressed as the percentage of the biomass of RO, GO, and SO in the total biomass of ramet, respectively. Unfortunately, the plant aerial part samples collected on August 13 were lost due to unexpected reasons; we missed some data such as biomass allocation, R/S ratio, and N from the sampling date.

In this study, these 12 traits (i.e., CGA_H_, CGA_D_, RGR_H_, NPR, LA, SLA, TNC, N, R/S, SRA, GA, and SA) were employed to calculate the overall GTI in order to reflect the effects of various potential mechanisms on grazing tolerance as much as possible. Some of these have been widely recognized in many previous studies, for example, increased NPR after damage, high RGR, increased ramet density, high levels of TNC in roots, and increased R/S ratio (Chapin & McNaughton, 1989; Strauss & Agrawal, 1999; Tiffin, 2000). Growth height (H), leaf area (LA), specific leaf area (SLA), and biomass allocation, which were often used in the study of *E. nutans* (Liu et al., 2009, 2005; Wu, Shen, Zhang, & Fu, 2009; Zhu et al., 2005), have been proposed as tolerance mechanisms by many researchers (Anten et al., 2003; Caldwell, Richards, Johnson, Nowak, & Dzurec, 1981; Damhoureyeh & Hartnett, 2002; Gao, Wang, Ba, Bai, & Liu, 2008; Ruess, McNaughton, & Couhghenour, 1983; Thompson, Cunningham, Ball, & Nicotra, 2003). In our study, the CGA_AB_ was used to indicate the compensatory growth ability. The GTI of each trait was obtained separately using the following formula:

(4) GTI=(initial value-induced value)×100/initial value

The initial value here refers to the trait value of unclipped, no fertilizing, and no watering treatment (i.e., control). We also calculated the GTI of CGA_AB_ in order to compare with the overall GTI.

To compare the variability of different traits on the same scale, we used relative range (RR) to express the response of a trait to defoliation damage, which equals the standardized induced value with the highest variation of a trait across treatments subtracted its standardized initial value.

### 2.4 Data analysis

We conducted statistical analyses using SPSS version 13.0 (SPSS, Chicago, IL). To determine whether clipping (C), fertilization (F), watering (W), and their interactions had any impact on these traits, we separately performed a mixed‐effects model for each trait using three‐way split‐plot ANOVAs, where block (B) was considered as random factor, C (whole plot factor), F, W, and their interactions (subplot factors) as fixed factors. The C effect (*df* = 2) was tested over the C × B interaction (*df* = 6); it is necessary to assume that there was no interaction between F or W and B, and the F effect (*df* = 1), the W effect (*df* = 1), the interaction of C × *F* (*df* = 2), C × W (*df* = 2), F × W (*df* = 1), and C × F × W (*df* = 2) were tested over the error term.

To increase normality and homogeneity of variance, the TNC, N, LA, CGA_D_, and CGA_AB_ were log‐transformed, and the RGR_H_, R/S, SA, SLA, and CGA_H_ were Blom‐transformed with SPSS 13.0 before analysis. Other traits were in compliance with normal distribution except for SRA which was analyzed by Kruskal–Wallis test. Where appropriate, analyses were followed by a multiple comparisons of means using a Tukey's post hoc tests. Differences in the above analyses were considered significant at *p < *0.05.

To examine the effective predictors and their relative contribution to grazing tolerance under varying conditions, we generated multiple linear regression models using overall GTI and GTI of CGA_AB_ as response variables, respectively, and GTI of the above 12 traits as explanatory variables, and evaluated these models using a model selection approach with Akaike information criterion (AICc) to select the best‐performing model. Because the overall GTI, GTI of CGA_AB _and GTI of each trait were relative measures, these explanatory variables were expressed on the same scale as response variables. A total of 12 stepwise regression analyses were carried out for the two expressions of tolerance. Two regressions were run separately for treatments MC and HC with different levels of fertilization and watering, two for treatments NF and F with different levels of clipping and watering, and two for treatments NW and W with different levels of clipping and fertilization, respectively. Multiple co‐linearity among traits was checked with variance inflation factor (VIF < 10, accepted) (O'Brien, 2007). For GTI calculating and stepwise regression analysis, data of RGR_H_, LA, SLA, NPR, CGA_H_, CGA_D_, and CGA_AB_ were year‐averaged, while TNC, N, GA, SRA, SA, and R/S were based on the data in August 23, 2009. The entrance significance level of predictor for the *F* statistic was set to *p < *0.05.

## 3 RESULTS

### 3.1 Traits response

**Figure 3:**
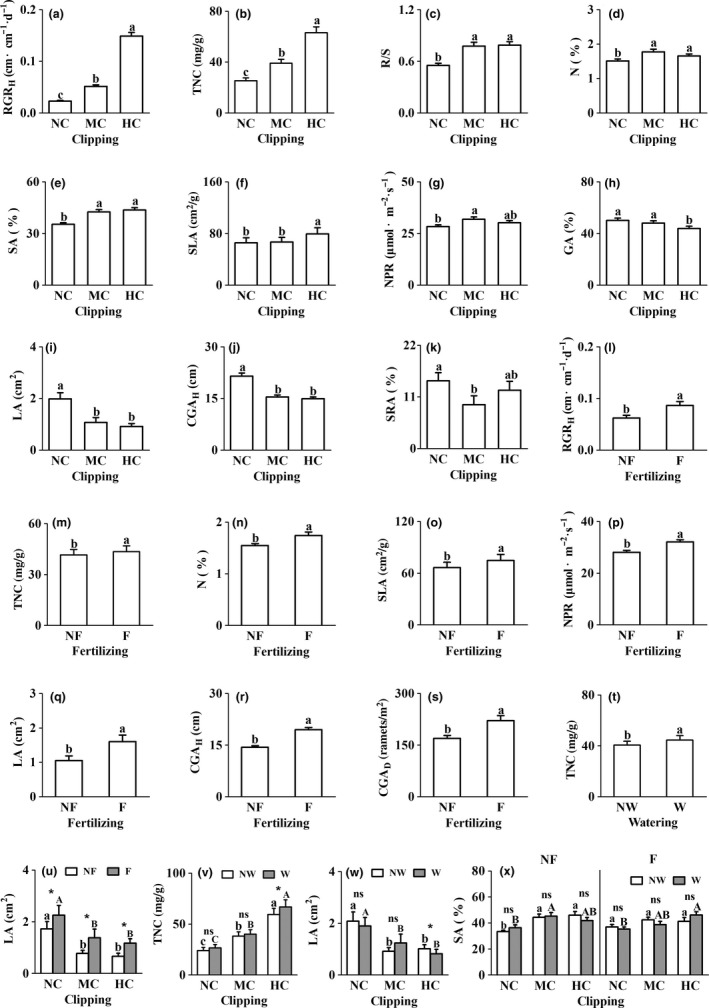
The responses (mean ± 1*SE*) of 12 traits in *Elymus nutans* ramet to different clipping, fertilization, watering treatments, and their interactions. F, fertilized; HC, heavy clipping; MC, moderate clipping; NC, nonclipping; NF, non‐fertilized; NW, non‐watered; W, watered. The abbreviations are the same as those in Table 1. The different letters above error bar indicated significant differences across treatments (*p < *0.05). “ns” or “*” indicated no significant or significant interaction between treatments (*p < *0.05), respectively

The 12 traits in this study showed a complex response pattern to experimental treatments (Table 1). In general, there were seven traits (i.e., RGR_H_, TNC, R/S, N, SA, SLA, and NPR) were increased after clipping (Figure 3a–g), four traits (i.e., GA, LA, CGA_H_, and SRA) were decreased (Figure 3h–k), and one trait (i.e., CGA_D_) showed no response to clipping (Table 1; Figure was not shown); eight traits (i.e., RGR_H_, TNC, N, SLA, NPR, LA, CGA_H_, and CGA_D_) were increased by fertilization (Figure 3l–s) except for four biomass allocation traits (i.e., R/S, GA, SA, and SRA) that did not respond to fertilization (Table 1; Figure was not shown). There were no significant differences in eleven traits between watering and nonwatering (Table 1; Figure was not shown), except TNC was increased after watering (Figure 3t).

**Table 1:** UNIANOVA for the effects of different treatments and their interactions on (a) the traits and (b) the compensatory growth of aboveground biomass of ramet in *Elymus nutans*

	Clipping (C)		Fertilization (F)		Watering (W)		C × F		C × W		F × W		C × F × W	
	*F*‐value	*p*‐Value	*F*‐value	*p*‐Value	*F*‐value	*p*‐Value	*F*‐value	*p*‐Value	*F*‐value	*p*‐Value	*F*‐value	*p*‐Value	*F*‐value	*p*‐Value
(a) Traits														
RGR_H_	821.408	0.000**	105.719	0.000**	0.503	0.480	0.947	0.391	0.725	0.487	0.072	0.789	0.200	0.819
TNC	552.292	0.000**	28.871	0.000**	109.893	0.000**	2.054	0.137	5.356	0.007**	0.895	0.348	1.612	0.208
R/S	9.242	0.002**	1.888	0.175	0.098	0.756	1.878	0.163	0.312	0.733	0.531	0.469	3.001	0.058
GA	7.142	0.006**	0.216	0.644	1.194	0.279	2.131	0.129	1.720	0.189	0.835	0.365	0.206	0.815
N	10.521	0.001**	14.785	0.000**	1.055	0.309	0.059	0.943	1.250	0.295	0.367	0.547	2.985	0.059
SA	9.731	0.002**	1.177	0.283	0.001	0.980	2.148	0.127	0.196	0.823	0.274	0.603	4.241	0.019*
SLA	5.859	0.021*	5.363	0.026*	0.138	0.712	2.431	0.102	0.647	0.530	0.856	0.361	0.567	0.572
NPR	5.492	0.025*	19.290	0.000**	0.614	0.439	3.077	0.058	0.555	0.579	2.498	0.123	2.110	0.136
LA	19.406	0.000**	63.550	0.000**	1.766	0.192	3.574	0.038*	6.051	0.005**	2.213	0.146	1.537	0.229
CGA_H_	40.401	0.000**	233.980	0.000**	0.633	0.428	0.175	0.839	0.797	0.453	0.069	0.793	0.777	0.462
CGA_D_	1.792	0.182	39.273	0.000**	0.937	0.335	0.700	0.499	2.092	0.128	0.021	0.885	0.313	0.732
^c^SRA	8.035	0.018*	0.751	0.386	0.611	0.434								
(b) Compensatory growth														
CGA_AB_	18.489	0.000**	90.733	0.000**	7.050	0.010*	1.904	0.159	2.218	0.119	0.543	0.464	0.280	0.757

Note

*p < 0.05, **p < 0.01. RGR_H_ is relative growth rate of ramet height; R/S is the ratio of root biomass to aboveground biomass per ramet; TNC is the total nonstructural carbohydrates of storage organs; SRA, GA, and SA refer to the allocation of total biomass per ramet to sexual reproductive, growth, and storage organs, respectively; N is the nitrogen content in leaves and culms of ramet; LA and SLA are leaf area and specific leaf area, respectively; NPR is lamina net photosynthesis rate; CGA_H_, CGA_D,_ and CGA_AB_ indicated compensatory growth ability of height, density, and aboveground biomass of ramet, respectively. ^c^SRA was analyzed by Kruskal–Wallis test.

The interaction between clipping and fertilization had no significant effect on all traits except LA that was increased at any clipping level after fertilization compared with no fertilization (Table 1; Figure 3u). Compared with nonwatering, watering only increased TNC and reduced LA in heavy clipping treatment (Figure 3v,w). The interaction between fertilization and watering had no significant effect on all traits, and the interaction among clipping, fertilization, and watering had only significant effect on SA (Table 1). Whether fertilization or not, watering had no significant effect on SA (Figure 3x). Under NF–NW, NF–W, and F–W treatments, SA was increased after clipping while showed no response to clipping in F–NW treatment (Figure 3x).

### 3.2 Changes of CGA_AB_

**Figure 4:**
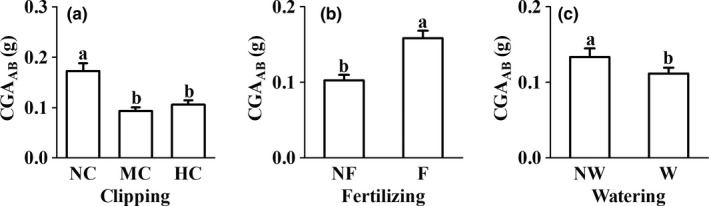
Changes in compensatory growth ability of aboveground biomass of ramet (CGA_AB_, mean ± 1*SE*) in *Elymus nutans* under different treatments. The abbreviations are the same as those in Figure 3. The different letters above error bar indicated significant differences across treatments (*p < *0.05)

The CGA_AB_ was decreased by clipping and watering, but was increased by fertilizing (Figure 4a–c). No significant interaction effects of these treatments on CGA_AB_ were found (Table 1).

### 3.3 Multiple co‐linearity among the traits

We found the redundant response of traits to experimental treatments because the correlation coefficient between four pairs of traits was greater than 0.8 under certain conditions (Table 2). In these four pairs of traits, SA and R/S showed redundant relationships in all treatments. The redundancy relationship between RGR_H_ and the other two traits, TNC and CGA_H_, was found in two treatments, respectively. The redundancy between GA and N existed in only one treatment (Table 2).

**Table 2:** Multiple co‐linearity between traits under different treatments

Paired traits	Correlation coefficients					
	MC	HC	NF	F	NW	W
SA vs. R/S	+0.976**	+0.948**	+0.945**	+0.986**	+0.963**	+0.933**
RGR_H_ vs. TNC			+0.804**		+0.821**	
RGR_H_ vs. CGA_H_	+0.960**	+0.969**				
GA vs. N	+0.832**					

Note

**p < 0.01. The meanings of abbreviations are similar as in Table 1 and Figure 3.

### 3.4 Tolerance expression

#### 3.4.1 Overall GTI

Based on the overall GTI,* Elymus nutans* showed over‐compensatory pattern, because the overall GTI calculated for 12 traits showed a 52.88% increment (i.e., overall GTI = −52.88) in mean performance in response to experimental conditions. Moreover, compared with the control (i.e., nonclipping, nonfertilization, and nonwatering), the average performance of the traits was increased in all other treatments and had a compensation capacity rank of HC (−88.80) > F (−55.88) > W (−48.53) > NW (−47.16) > NF (−39.81) > MC (−37.08). We calculated the increased/decreased ratio between the treatments according to the calculation: (value in treatment 1 − value in treatment 2) × 100/value in treatment 2. The average compensation capacity in treatment HC was 139.5% higher than that in MC along the gradient of resource availability. On the clipping gradient, fertilization and watering increased the average compensation capacity by 40.4% and 2.9%, respectively, compared with nonfertilization and nonwatering treatment (Table 3).

**Table 3:** Two expressions of tolerance based on the overall GTI of 12 traits and the compensatory growth of ramet of *Elymus nutans* under different clipping intensities and resource availabilities

Putative mechanisms and expression of tolerance	RIV	RR	Mean GTI	Grazing tolerance index (GTI) values of putative mechanisms under varying experimental conditions						Frequency as an effective predictor in all treatments	
				Clipping level		Fertilizing level		Watering level			
				MC	HC	NF	F	NW	W	Overall GTI	GTI of CGA_AB_
Putative mechanisms											
RGR_H_	0.128	0.872	−337.72	−173.68	−684.21	−226.32	−357.90	−300.00	−284.21	5	1
TNC	0.385	0.615	−92.63	−72.43	−160.02	−76.24	−85.43	−71.72	−89.94	4	0
R/S	0.399	0.601	−113.27	−106.36	−150.84	−135.70	−75.51	−102.62	−108.60	6	1
GA	0.584	0.416	−65.39	−71.13	−56.03	−62.58	−70.01	−62.31	−70.28	5	0
N	0.604	0.396	−54.53	−65.53	−57.98	−51.04	−50.79	−45.31	−56.52	4	1
SA	0.637	0.363	−46.16	−45.92	−56.98	−51.35	−35.68	−43.20	−43.83	0	0
NPR	0.805	0.195	−17.69	−23.51	−16.52	−8.86	−24.18	−17.89	−15.15	5	2
SLA	0.816	0.184	−10.39	−3.41	−22.59	−2.68	−15.49	−9.05	−9.11	4	1
CGA_H_	0.901	−0.157	6.70	12.01	15.39	17.40	−11.01	2.23	4.15	4	4
CGA_D_	0.928	−0.161	6.18	4.82	12.85	17.41	−7.72	3.67	6.03	4	0
LA	1.000	−0.490	32.33	40.13	49.03	41.52	10.89	25.57	26.85	5	2
SRA	1.000	−0.623	58.07	60.07	62.33	60.75	52.26	54.77	58.24	0	0
Overall GTI			−52.88	−37.08	−88.80	−39.81	−55.88	−47.16	−48.53		
Grazing tolerance rank^a^				6	1	5	2	4	3		
GTI of CGA_AB_			22.26	37.58	28.86	36.91	−3.36	9.40	24.16		
Grazing tolerance rank^b^				6	4	5	1	2	3		

Note

RIV is the relative initial value of traits (i.e., measure under unclipped, no fertilization, and no watering); RR refers to the relative range of traits, the minus sign before RR indicates that the induced value is less than the initial value. The meanings of abbreviations are similar as in Table 1 and Figure 3.

a Rank based on overall GTI.

b Rank based on GTI of CGA_AB_.

#### 3.4.2 GTI of CGA_AB_

Compared with the over‐compensatory pattern based on overall GTI of 12 traits, aboveground biomass of ramet was an under‐compensatory pattern because its performance showed an average reduction of 22.26% (i.e., mean GTI of CGA_AB_ = 22.26) in its response to treatments. And it was reduced in all other treatments except for treatment F. The rank of its across treatments was F (−3.36) > NW (9.40) > W (24.16) > HC (28.86) > NF (36.91) > MC (37.58). It was 23.2% higher in treatment HC than in treatment MC along the gradient of resource availability. Compared with nonfertilization and nonwatering, fertilization increased it by 109.1% and watering decreased 157.0%, respectively, on the clipping gradient (Table 3).

### 3.5 Variability of traits

Pearson product‐moment correlation showed that the relative initial value (RIV) of traits was negatively correlated with the relative range (RR) (*r *= −0.939, *p* < 0.001, *n* = 12) (Table 3). The smaller the RIV was, the more negative the mean GTI (*r = *0.923, *p* < 0.001, *n* = 12); the greater the RR was, the more negative the mean GTI (*r *= −0.826, *p* = 0.001, *n* = 12) (Table 3). Because the absolute RR values were greater than 0.6, the RGR_H_, TNC, R/S, and SRA were considered as highly variable traits in the study, while NPR, SLA, CGA_H_, and CGA_D_ were less variable traits because their absolute RR values were less than 0.25. The absolute RR values of the remaining four traits (i.e., GA, N, SA, and LA) ranged from 0.25 to 0.60, which were considered to be moderately variable (Table 3).

### 3.6 Effective predictors of the expressions of tolerance

#### 3.6.1 Overall GTI

Ten effective predictors, that is, R/S, RGR_H_, LA, TNC, GA, NPR, SLA, N, CGA_D_, and CGA_H_, explained 97.3%–100% of the variance in overall GTI (Table 4). There are 5, 5, 6, 10, 10, and 10 effective predictors in treatment MC, HC, NF, F, NW, and W, respectively, and the average number of effective predictors across treatments was 7.7 (Table 4). The remaining traits, SA, and SRA were invalid predictors and were excluded from regression equations of all treatments.

**Table 4:** The stepwise regression equations for predicting two expressions of grazing tolerance of *Elymus nutans* in different experimental treatments with overall GTI or GTI of CGA_AB_ as the response variable (*Y*) and with the GTI of 12 traits as explanatory variables

Treatments		Expression based on the overall GTI						Expression based on the GTI of CGA_AB_					
		Standardized regression equations	AICc	*F*	*R* ^2^	*p*	*df* (*i*, *j* )	Standardized regression equations	AICc	*F*	*R* ^2^	*p*	*df* (*i*, *j*)
Clipping	MC	*Y* = 0.517 LA + 0.426 CGA_D_ + 0.322 R/S + 0.287 N + 0.253 NPR	69.93	111.00	0.973	<0.001	5,10	*Y* = 0.886 CGA_H_	105.89	51.29	0.770	<0.001	1,14
	HC	*Y* = 0.634 RGR_H_ + 0.554 R/S + 0.276 LA + 0.189 TNC + 0.086 GA	71.48	183.20	0.984	<0.001	5,10	*Y* = 0.690 LA + 0.315 NPR	96.69	19.07	0.707	<0.001	2,13
Fertilizing	NF	*Y* = 0.733 RGR_H_ + 0.337 R/S + 0.204 GA + 0.083 SLA + 0.074 NPR + 0.050 CGA_H_	90.47	515.50	0.993	<0.001	6,17	*Y* = 0.644 CGA_H_ − 0.300 N	172.98	23.08	0.658	<0.001	2,21
	F	*Y* = 0.750 RGR_H_ + 0.188 TNC + 0.182 R/S + 0.177 LA + 0.122 GA + 0.096 SLA + 0.095 CGA_D_ + 0.082 N + 0.060 NPR + 0.055 CGA_H_	−33.68	1.44 × 10^5^	1.000	<0.001	10,13	*Y* = −0.693 RGR_H_ + 0.565 R/S	175.19	14.00	0.531	<0.001	2,21
Watering	NW	*Y* = 0.652 RGR_H_ + 0.233 R/S + 0.144 TNC + 0.112 LA + 0.091 CGA_D_ + 0.087 GA + 0.075 N + 0.055 SLA + 0.055 NPR + 0.048 CGA_H_	−19.84	9.84 × 10^4^	1.000	<0.001	10,13	*Y* = 0.711 CGA_H_ − 0.345 SLA + 0.253 NPR	189.09	11.30	0.573	<0.001	3,20
	W	*Y* = 0.725 RGR_H_ + 0.336 R/S + 0.212 TNC + 0.204 LA + 0.132 GA + 0.124 SLA + 0.102 N + 0.102 CGA_D_ + 0.072 CGA_H_ + 0.051 NPR	4.49	2.42 × 10^4^	1.000	<0.001	10,13	*Y* = 0.659 CGA_H_ + 0.274 LA	161.39	25.67	0.682	<0.001	2,21

Notes

The meanings of abbreviations are similar as in Table 1 and Figure 3.

*i* and *j* represent regression and residual degrees of freedom, respectively.

Among the effective predictors, R/S entered into the prediction equations of all treatments and was the most common effective predictors. RGR_H_, GA, LA, NPR, TNC, N, SLA, CGA_D, _and CGA_H_ entered into five or four equations of them and were the second most common predictors (Tables 3 and 4). RGR_H_ had the largest relative contribution to overall GTI, with a range of variation in standardized regression coefficients (SRCs) from 0.634 to 0.750 across treatments except in treatment MC (Table 4).

#### 3.6.2 GTI of CGA_AB_

In total, there were seven effective predictors for GTI of CGA_AB_ across treatments, that is, CGA_H_, LA, SLA, NPR, RGR_H_, R/S, and N; these predictors explained 53.1%–77.0% of the variance in GTI of CGA_AB _(Table 4). The other five traits, TNC, GA, CGA_D_, SA, and SRA were invalid predictors (Table 4). The number of effective predictors included in treatment MC, HC, NF, F, NW, and W was 1, 2, 2, 2, 3, and 2, respectively, with an average number of 2 per treatment. Among them, CGA_H_ entered into the prediction equations of four treatments and was the most common effective predictor. LA and NPR entered into two equations of them and were the second most common predictors. RGR_H_, R/S, N, and SLA entered into one of them and were the less common effective predictors (Tables 3 and 4). Meanwhile, CGA_H_ had the largest relative contribution to GTI of CGA_AB_, with a range of variation in SRCs from 0.644 to 0.886 except for treatment HC and F (Table 4).

## 4 DISCUSSION

### 4.1 Compensatory growth ability

Our results clearly showed that *E. nutans* had limited compensatory growth capacity because, although clipping induced an increase in the performance of some traits (Figure 3a–g), the CGA_AB_ of both clipped treatments was always significantly lower than that of unclipped treatment (Figure 4a). This can be attributed to the decline in the capture rate of carbon assimilates due to the decrease of aboveground allocation (GA and SRA, Figure 3h,k), leaf area (LA, Figure 3i), and plant height (CGA_H_, Figure 3j) after clipping. This pattern of response to clipping damage is in agreement with observations in many studies on grass species (Anten et al., 2003; Damhoureyeh & Hartnett, 2002; Gao et al., 2008; Li et al., 2016; Liu et al., 2005; Pankoke & Müller, 2013; Strauss & Agrawal, 1999; Zhao, Chen, Han, & Lin, 2009; Zhao et al., 2008; Zhu & Sun, 1996). As in previous studies (Gao et al., 2008; Gerdol, Brancaleoni, Marchesini, & Bragazza, 2002; Gough et al., 2012; Hicks & Turkington, 2000; Jiang, Dong, Gan, & Wei, 2005; Kohyani et al., 2009; van Staalduinen, Dobarro, & Peco, 2010; Wang et al., 2003), many traits in the study, including CGA_AB_, showed significant increases after fertilization (Figures 3 and 4), suggesting nitrogen is a focal resource limiting plant growth (van Staalduinen et al., 2010; Wise & Abrahamson, 2007). According to the limiting resource model (LRM, Wise & Abrahamson, 2007), the types of focal resources are different before and after defoliation damage, with nitrogen before damage and carbon after damage. In this study, the exacerbating effect of the defoliation on carbon limitation was not mitigated by fertilization, because the carbon allocation in the aboveground part of the species did not increase significantly after fertilization (Table 1), as observed by Wu et al. (2009). Therefore, this study suggests that rich‐nutrient conditions may have only a limited positive effect on grazing tolerance of the species, which partly supports the antagonistic interaction hypothesis between defoliation damage and nutrient availability (Gao et al., 2008).

Both the growth rate model (GRM) (Hilbert et al., 1981) and the continuum of responses hypothesis (CRH) or the compensatory continuum hypothesis (CCH) (Huhta, Hellström, Rautio, & Tuomi, 2000; Maschinski & Whitham, 1989) emphasized the importance of growth rate or photosynthetic rate after defoliation damage to compensatory growth. However, in our study, although the NPR (Figure 3g,p) and the RGR_H_ (Figure 3a,l) increased significantly after clipping and/or fertilization, the low CGA_AB_ (Table 3) clearly suggested that the increases in performance of these physiological traits did not effectively improve grazing tolerance as expected, as some studies have shown (Hilbert et al., 1981; Oesterheld & McNaughton, 1991; Tiffin, 2000; Zhao et al., 2008). We believe that the prerequisite for increased physiological response after defoliation damage to promote grazing tolerance is that sufficient storage resources must be maintained in plant roots. If the storage resources are limited, as the trade‐offs theory of energy allocation predicts, the allocation of resources to other functions is inevitably reduced when the physiological response increases, and therefore, the grazing tolerance will not increase significantly. Therefore, the results of this study provided only limited support for the CRH and CCH predictions.

Under the influence of successive years of clipping stress, GA, LA, CGA_H_, and SRA (Figure 3h–k) decreased, while R/S (Figure 3c), SA (Figure 3e), and TNC (Figure 3b) increased. This reflected the species’ strategy for self‐protection and avoidance of defoliation‐induced mortality against defoliation damage because plants could adapt disturbance by increasing the storage biomass allocation and reducing energy consumption, leading to lower edible rate and energy loss rate, only then can ensure the growth of vegetative and reproductive branches and then reduce the negative influence of grazing and abnormal climate conditions to population growing (Wei, Yan, Yun, Chu, & Yang, 2011). Some studies have shown that rich‐nutrient conditions are particularly important to improve plant grazing tolerance in cold environments (Chapin & McNaughton, 1989; Coughenour, McNaughton, & Wallace, 1985). In the present study, clipping decreased SRA (Figure 3k) but not the CGA_D_ (Table 1), whereas fertilization increased CGA_D_ (Figure 3s) but not SRA (Table 1), suggesting that under the cold environments in the alpine meadow, it might be more important for plant to respond through mechanisms that enable them to survive rather than through mechanisms that would safeguard reproduction when they are damaged. Therefore, nutrient has significantly improved the vegetative propagation capacity of plants, and nutrient supplementation may play an important role in the long‐term maintenance of the species.

Other eleven traits were not responsive to watering (Table 1), except TNC was increased after watering (Figure 3t). Therefore, compared with fertilization, the effect of watering on improving tolerance is very limited although the average annual precipitation during the study period was lower than that of long‐term precipitation. Moreover, the GTI of CGA_AB_ decreased by 157.0% after watering compared with that without watering (Table 3), which confirmed the cooperative interaction of defoliation damage and water availability on grazing tolerance (Gao et al., 2008).

### 4.2 Two opposite expressions of tolerance

Our results showed that the grazing tolerance expression of overall GTI based on 12 traits was completely opposite to that based on CGA_AB_ because of the former was over‐compensation and did not vary with clipping intensity and resource availability, while the latter was under‐compensation and only converted to limited over‐compensation after fertilization (Table 3). This indicates that the expression of grazing tolerance based on overall GTI not only did not approximately reflect the characteristics of the species' limited compensatory growth capacity, but also greatly overestimated this capacity. Therefore, our results negate the first assumption implied in the overall GTI, and indicate that the grazing tolerance of plants should not be regard as a multivariate linear function of traits considered; otherwise, the two expressions based on overall GTI and CGA_AB_ should be the same rather than the opposite.

Why were there two opposite expressions of tolerance in the same species? We think that four plausible causes may explain this phenomenon. Firstly, our data showed that overall GTI was strongly influenced by the variability of traits. On one hand, the more variable the trait, the greater the absolute value of its mean GTI. On the other hand, RGR_H_, TNC, and R/S, which were the common effective predictors and the bigger relative contributors for predicting changes in overall GTI were highly variable traits with extremely high negative mean GTI (Tables 3 and 4); the summed mean GTI of these traits greatly inflated the overall GTI and resulted in an over‐compensation pattern of overall GTI (Table 3). These results suggest that overall GTI is actually a measure of trait variability. However, according to a study by Wise et al. (2008), although some traits do not have high plasticity, they are important mechanisms of tolerance, and plasticity is not necessarily proportional to the effect of traits on tolerance. For example, in our study, less variable trait CGA_H _(RR = −0.157) was the most common effective predictor and the biggest relative contributor of the CGA_AB_ (Tables 3 and 4). Although some studies have shown that the initial or induced values of traits are closely related to plant tolerance (Hilbert et al., 1981; Suwa & Maherali, 2008; Wise et al., 2008), however, our results show that the variability or plasticity of traits cannot be regarded as the sole criterion for determining whether a trait is an important mechanism for grazing tolerance. The second hypothesis implied in the overall GTI is therefore denied. Our results also emphasize that if the overall GTI is used to characterize the grazing tolerance, the over‐expression of highly variable traits can greatly exaggerate the compensatory response and inflate the overall GTI, which may lead to a large deviation between the overall GTI and the actual compensatory ability, and thus misestimate the grazing tolerance of plants.

Secondly, the overall GTI can not accurately reflect the response of traits to experimental treatments, which is related to the fact that it is impossible to exclude the random errors contained in trait responses when calculating the GTI of traits. In this study, the numbers of such traits with no significant difference (*p* > 0.05) in observed values among different levels of clipping, fertilization, and watering were 1.0 (i.e., CGA_D_), 4.0 (i.e., R/S ratio, GA, SA, and SRA), and 11.0 (i.e., RGR_H_, R/S ratio, GA, N, SA, NPR, CGA_D_, LA, SLA, CGA_H_, and SRA), respectively (Table 1). For example, the effect of fertilization on the R/S ratio was statistically insignificant along the clipping intensity gradient (Table 1); the difference of 1.34 times in observed values for this trait between fertilized (0.94 ± 0.01) and unfertilized plants (1.26 ± 0.03) was generally regarded as the result of random error. However, the GTI of this trait differed by 1.81 times between fertilized (−75.51) and unfertilized plants (−135.70) (Table 3), and was directly used to calculate the overall GTI of the two treatments together with the other 12 traits. This means that the more such traits considered, the greater the additive effect of the random errors they contain on the overall GTI, and the more likely it is that there will be a greater deviation between the two expressions of tolerance. Therefore, in this sense, it may be more accurate to use the single trait aboveground biomass to evaluate the grazing tolerance, because the variation of it is the final result of multitrait response (Lepš et al., 2006; Tiffin, 2000). The response of multiple traits may be more suitable to reveal the possible mechanisms promoting tolerance to herbivory.

Thirdly, the doubling weight of functionally redundant traits is also one of the reasons resulting in over‐compensation pattern in overall GTI. Because the functional redundancy of two traits means that the ecological function represented by one trait can be replaced by another (Lepš et al., 2006) and the overall GTI is equal to the arithmetic average of the GTI of the traits considered, if a pair of redundant trait is included in the calculation of the overall GTI, it actually means that the weight of a specific ecological function is doubled. This not only overestimates the overall GTI, but also increases the degree of overestimation with the increase of the number of pairs of redundant traits. In this study, total 4 pairs of traits that combined by 7 traits showed redundant relationship (Table 2), and 6 of the 7 traits (except CGA_H_) showed increased response to each treatment (Table 3). In addition, there was a pair of redundant traits at least being in all treatments, and even three pairs of such traits in some treatments (Table 2). Therefore, the overall GTI of each treatment shown in Table 3 is actually the result of doubling weight of each pair of redundant traits. This may be another important reason why grazing tolerance expression based on overall GTI is obviously superior to that based on CGA_AB_. In the present study, we could not judge how many and which traits should be used, how to weight them and how to combine traits to quantify a species’ overall GTI more reasonably. In spite of this, the present results provide sufficient grounds to conclude that a simple linear combination based on multitrait responses, such as overall GTI, may not be an ideal estimator for compensatory growth capacity although considering the response of multiple traits may be a fundamental question for both theoretical and empirical studies of grazing tolerance.

Fourthly, the determining mechanisms of the two expressions of tolerance are different because they differ in terms of the composition of effective predictors, the most common effective predictors, and their relative contributions (Table 4). For example, although the two expressions of tolerance shared a total of seven effective predictors across treatments, for a particular treatment, they shared only a few effective predictors, the number of which was 0, 1, 1, 2, 3, and 2 in treatment MC, HC, NF, F, NW, and W, respectively (Table 4). This suggests that the underlying mechanisms that determine the overall GTI could not predict the changes in CGA_AB_. Instead, as this study shown, we can predict CGA_AB_ with fewer and more efficient predictors.

We believe that the traits used to understand or predict a plant's grazing tolerance should meet at least three criteria: universality, they can be effective predictors under various grazing disturbances and resource availability conditions; predictability, they have higher relative contribution to grazing tolerance; simplicity but completeness, selected trait set should be able to reflect different aspects of potential mechanism of grazing tolerance through as few traits as possible. Based on our research to *E*.* nutans* (Table 4), we consider CGA_H_ to be the preferred trait, because it is not only the most common effective predictor of aboveground biomass, but also the trait that contributes most to aboveground biomass in most treatments, which meets the first two criteria. Furthermore, LA and RGR_H_ are also optional traits because they have the most relative contribution to aboveground biomass in a certain treatment, respectively (Table 4). For the third criterion, we consider that another four effective predictors, that is, NPR, R/S, SLA, and N (Table 4), are also available for selection. However, as simplicity means minimal trait redundancy, we have selected two traits from them according to the mechanisms that have been widely recognized at present (Chapin & McNaughton, 1989; Strauss & Agrawal, 1999; Tiffin, 2000), including NPR and R/S. Finally, we recommend a trait set consisting of five traits, namely CGA_H_, LA, RGR_H_, NPR, and R/S in order to explain the grazing tolerance of this species. Nevertheless, we also think that this trait set should be species dependent and disturbance dependent, and it is not excluded that there are other trait sets under other environmental conditions and for different species.

## 5 CONCLUSIONS

Because the expression of grazing tolerance based on the overall GTI cannot truly reflect the characteristics of the limited compensatory growth ability of *E. nutans*, but drastically overestimates this ability, the mechanism that determines the overall GTI cannot predict the change of the compensatory growth of the species also, we therefore consider that overall GTI is not an ideal predictor for describing the single‐species tolerance to grazing. It may be suitable for using the same set of traits to assess differences in grazing tolerance among more than two species because, in this case, factors that could inflate overall GTI (i.e., the over‐expression of highly variable traits with extremely high negative mean GTI, the random errors contained in traits considered and the doubling weight of redundant traits) may be eliminated as systematic errors. Our data suggest that plant tolerance to grazing is not a multivariate linear function of investigated traits or mechanisms, but mainly depend on the relative contribution of traits with lower variable to defoliation damage.

## CONFLICT OF INTEREST

The authors declare no competing financial interests.

## AUTHOR CONTRIBUTIONS

Z‐H.Z. designed the experiment and conceived the idea of this study. All authors performed the field experiment. L‐L.Z. conducted laboratory work and analyzed the data. Z‐H.Z. and L‐L.Z. performed the literature search. The manuscript is written by L‐L.Z. and modified by Z‐H.Z, Z‐Q.Q and G.L.

## Data Availability

The data are provided in Supporting Information Appendix [Link], [Link], [Link], [Link], [Link].
